# Analysis of duodenal ulcer mortality trends by sex, race, age, and region in the United States, 1999–2023: A CDC WONDER database study

**DOI:** 10.1097/MD.0000000000050055

**Published:** 2026-08-28

**Authors:** Ming-Jie Jia, Bo-Ying Wu, Feng Jiang

**Affiliations:** a The Second Affiliated Hospital of Guangxi University of Chinese Medicine, Nanning, Guangxi Zhuang Autonomous Region, China.

**Keywords:** CDC WONDER, duodenal ulcer, health inequities, Joinpoint regression, mortality

## Abstract

To systematically evaluate long-term trends in duodenal ulcer (DU)-related mortality in the United States (US) from 1999 to 2023 using US Centers for Disease Control and Prevention, Wide-ranging Online Data for Epidemiologic Research mortality registry data and to identify disparities by sex, race/ethnicity, age, region, and urban–rural setting, a retrospective population-based study design was employed. DU-related deaths were identified using International Classification of Diseases, 10th Revision code K26. Annual death counts were extracted, and age-adjusted mortality rates were calculated using the 2000 US standard population. Joinpoint regression (with Monte Carlo permutation testing) estimated annual percentage changes (APCs) and average APCs, reporting 95% confidence intervals. From 1999 to 2023, a total of 34,520 cumulative DU-related deaths occurred in the US The overall age-adjusted mortality rate declined from 0.83 to 0.64 per 100,000 (average APC = −1.32; 95% confidence interval = −2.75–0.14), with a marked decrease through 2009 followed by flattening and signs of resurgence thereafter. This “decline-then-rise” pattern was most evident among men, older adults (≥ 65 years), several racial and ethnic groups (including non-Hispanic White, Hispanic, and other races), and across all 4 census regions, while nonmetropolitan areas consistently had higher age-adjusted mortality rates than metropolitan areas. State-level analyses showed generally declining mortality with recent rebounds in several states. Over the past 2 decades, DU-related mortality in the US has generally declined. However, the rate of decline slowed around 2010, with rebounds occurring in certain populations. Persistent inequalities persist across sex, race/ethnicity, geographic regions, and urban/rural settings. Future public health strategies should implement stratified, targeted interventions for elderly and high-risk medication populations to reduce disparities and address emerging challenges.

## 1. Introduction

Duodenal ulcer (DU) is a common form of peptic ulcer disease and represents a mucosal defect resulting from the digestive action of gastric acid and pepsin on the duodenal mucosa. DU develops when the balance between mucosal aggressive factors (principally gastric acid and *Helicobacter pylori* infection) and mucosal defense and repair mechanisms is disrupted.^[1,2]^ Clinical presentation ranges from asymptomatic disease to food-related epigastric pain and may progress to life-threatening complications, including upper gastrointestinal hemorrhage, perforation, and pyloric obstruction.^[3,4]^ Since the identification of *H pylori* as the primary cause of peptic ulcer disease in 1982, management has shifted from acid suppression alone to comprehensive, etiology-directed strategies emphasizing eradication therapy.^[2]^ In parallel, widespread use of proton pump inhibitors (PPIs) and advances in endoscopic hemostasis have become cornerstones of DU and complication management.

Epidemiologically, *H pylori* infection remains the leading risk factor. The extensive use of nonsteroidal anti-inflammatory drugs (NSAIDs) and aspirin has emerged as an independent and increasingly important cause, particularly among older adults.^[5,6]^ Additional correlates include male sex, cigarette smoking, alcohol misuse, and psychosocial stress. Since the latter half of the 20th century, improved living conditions together with PPI adoption and endoscopic advances have been associated with overall declines in incidence, hospitalizations, and mortality across many developed countries.^[7]^ However, substantial heterogeneity persists across populations and regions: prognosis is closely linked to age, sex, socioeconomic status, and access to care, with older adults, rural residents, and certain racial and ethnic minority groups experiencing higher risk.^[3]^ Moreover, rising antimicrobial resistance in *H pylori* and prolonged NSAID/aspirin exposure in aging populations may attenuate earlier gains and contribute to adverse trends in specific subgroups.

In the United States (US), comprehensive analyses using a nationwide mortality registry to characterize long-term trends in DU-related deaths and to compare disparities by sex, race and ethnicity, age, region, and urban–rural residence remain limited. The Centers for Disease Control and Prevention, Wide-ranging Online Data for Epidemiologic Research (CDC WONDER) mortality platform provides standardized, nationwide coverage and enables calculation of age-adjusted mortality rates (AAMRs) standardized to the year 2000, allowing robust assessment of temporal changes in disease burden. This study uses CDC WONDER (International Classification of Diseases, 10th Revision [ICD-10] code K26) to evaluate overall trends and stratified disparities in DU-related mortality from 1999 to 2023 in the US, to identify high-risk populations and geographic hotspots, and to inform targeted clinical and public health strategies for prevention and resource allocation.

## 2. Methods

### 2.1. Study population and data source

We conducted a retrospective population-based study using the CDC WONDER Multiple Cause of Death database to identify all US decedents with DU listed between January 1, 1999, and December 31, 2023. DU-related deaths were ascertained by ICD-10 code K26. The death related to DUs as defined in this study refers to cases where the underlying cause of death is coded according to the ICD-10 as K26.0 to K26.9. These codes specifically correspond to DUs, including their acute, chronic, and unspecified types, as well as whether they are accompanied by complications such as bleeding or perforation. Gastric ulcers (coded K25.0–K25.9) are explicitly excluded from the scope of this study. The database has been used to evaluate mortality trends for various conditions (e.g., obesity and hypertension).^[8,9]^ Data extraction was limited to US resident deaths using default CDC WONDER categorizations; no additional recoding, filtering, or exclusions were applied. Mortality data derive from death certificates filed in the 50 states and the District of Columbia and exclude nonresident deaths (including foreign nationals, US citizens dying abroad, and residents of US territories) and fetal deaths.

### 2.2. Data extraction and variable definitions

Annual counts of DU-related deaths were obtained. AAMRs per 100 000 population were calculated by direct standardization to the 2000 US standard population. Denominator data for demographic strata were extracted as provided by CDC WONDER and included sex (male, female), race/ethnicity (Hispanic, non-Hispanic Black, non-Hispanic White, other), US Census region (Northeast, Midwest, South, West), age group (25–39, 40–54, 55–69, 70–84, ≥ 85 years), and urban–rural classification. Urban–rural status followed the National Center for Health Statistics scheme based on 2013 census-derived county classifications, grouped as metropolitan (population ≥ 50 000) and nonmetropolitan (population < 50 000).^[10]^ All definitions were consistent with those commonly used in CDC WONDER.^[11]^

### 2.3. Statistical analysis

We calculated crude mortality rates and AAMRs for each demographic stratum. Temporal trends in DU-related mortality from 1999 to 2023 were analyzed using Joinpoint regression (version 5.4.0; National Cancer Institute). We used the grid-search method, allowing from 0 to 3 joinpoints and requiring at least 2 yearly observations between 2 joinpoints and from any joinpoint to either end of the time series. Monte Carlo permutation tests were used to identify joinpoints. We estimated annual percent change (APC) for each segment and average APC (AAPC) for the entire study period, with 95% confidence interval (CIs). Two-sided *t*-tests assessed whether APCs/AAPCs differed from 0; *P* ≤ .05 was considered statistically significant. Statistically significant changes are indicated with asterisks in the Results. Due to the privacy-preserving suppression of small counts (typically < 10) in the CDC WONDER database, some subage group data are unavailable. Therefore, the trend analysis in this study is based on the complete data standard age groups.

This study used publicly available, de-identified data, adhered to the Strengthening the Reporting of Observational Studies in Epidemiology reporting guideline, and was exempt from institutional review board oversight under the US Common Rule (45 Code of Federal Regulations §46).

## 3. Results

### 3.1. Overall

**Figure 1.**
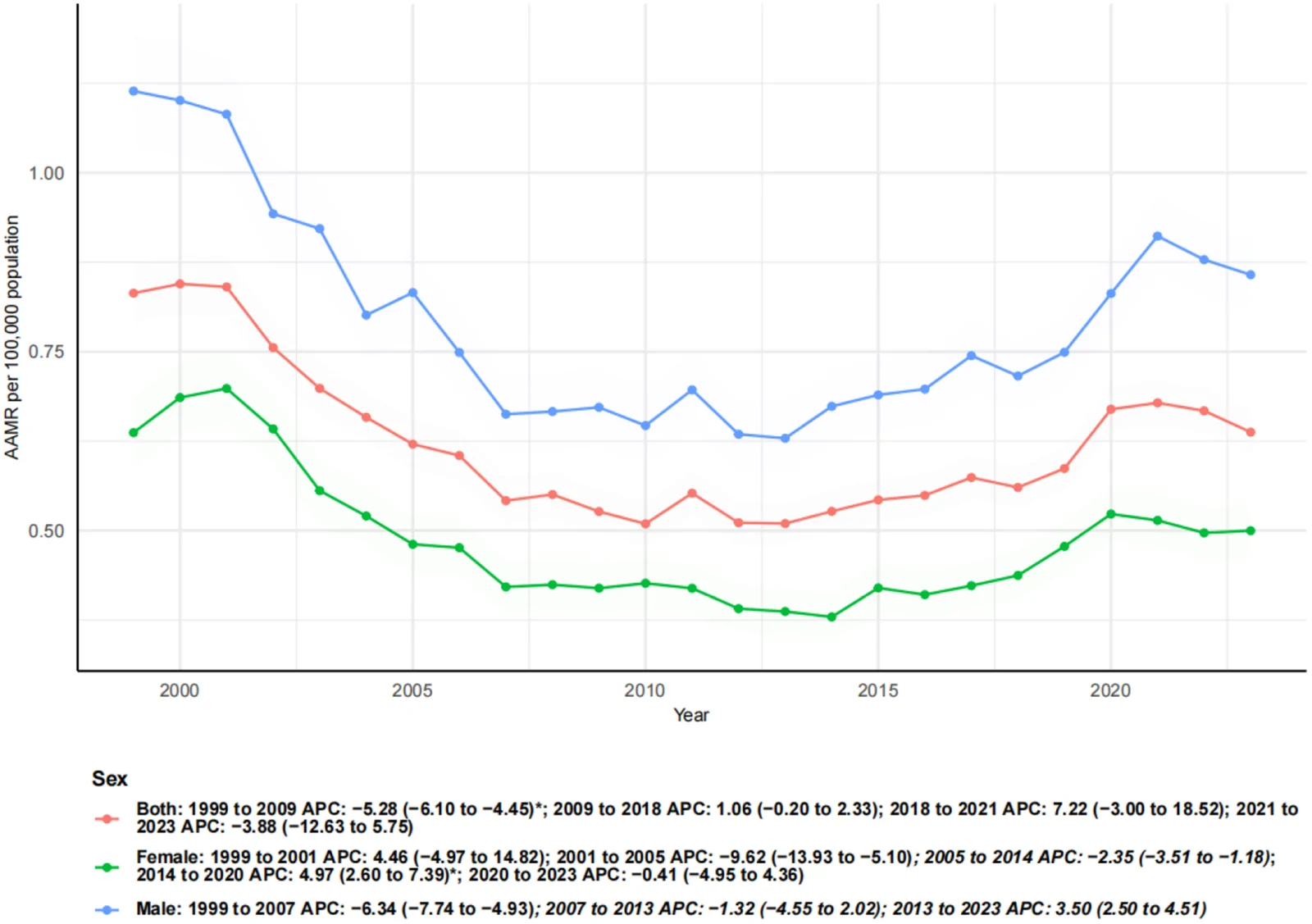
Trends in DU AAMRs stratified by sex in the US, 1999–2023. AAMR = age-adjusted mortality rate, APC = annual perventage change, DU = duodenal ulcer, US = United States.

From 1999 to 2023, there were 34,520 DU-related deaths in the US. The AAMR declined from 0.83 (95% CI: 0.79–0.87) per 100 000 in 1999 to 0.64 (95% CI: 0.61–0.67) in 2023. The AAPC was −1.32 (95% CI: −2.75–0.14), not statistically significant. Joinpoint regression identified a significant decline during 1999–2009 (APC = −5.28; 95% CI: −6.10–−4.45), followed by nonsignificant changes in 2009 to 2018 (APC = 1.06; 95% CI: −0.20–2.33), 2018 to 2021 (APC = 7.22; 95% CI: −3.00–18.52), and 2021 to 2023 (APC = −3.88; 95% CI: −12.63–5.75). See Table 1 and Figure 1 for details.

**Table 1:** Trends in DU AAMRs stratified by sex in the US, 1999–2023.

Sex	Deaths_1999	Deaths_2023	Percent. change	AAMR_1999	AAMR_2023	AAPC (95% CI)
Both	1470	1794	22.04	0.83 (0.79–0.87)	0.64 (0.61–0.67)	−1.32 (−2.75–0.14)
Female	680	756	11.18	0.64 (0.59–0.69)	0.50 (0.46–0.54)	−1.05 (−2.33–0.25)
Male	790	1038	31.39	1.11 (1.03–1.19)	0.86 (0.80–0.91)	−1.08 (−2.04–−0.10)*

AAMR = age-adjusted mortality rate, AAPC = average annual perventage change, CI = confidence interval, DU = duodenal ulcer, US = United States.

* indicates that the *P* value is statistically significant.

### 3.2. Population differences

#### 3.2.1. Sex

From 1999 to 2023, there were 34,520 cumulative DU-related deaths in the US: 18,799 (54.5%) among males and 15,721 (45.5%) among females. AAMRs were consistently higher in males. Among males, the AAMR declined from 1.11 (95% CI: 1.03–1.19) per 100,000 in 1999 to 0.86 (95% CI: 0.80–0.91) in 2023; among females, from 0.64 (95% CI: 0.59–0.69) to 0.50 (95% CI: 0.46–0.54). The AAPC indicated a significant decline in males (−1.08; 95% CI: −2.04–−0.10) but was not significant in females (−1.05; 95% CI: −2.33–0.25).

Joinpoint results showed, for males, a significant decline during 1999 to 2007 (APC = −6.34; 95% CI: −7.74–−4.93), no significant change in 2007 to 2013 (APC = −1.32; 95% CI: −4.55–2.02), and a significant increase in 2013 to 2023 (APC = 3.50; 95% CI: 2.50–4.51). For females, changes were not significant in 1999 to 2001 (APC = 4.46; 95% CI: −4.97–14.82), followed by a significant decline in 2001 to 2005 (APC = −9.62; 95% CI: −13.93–−5.10), a slower decline in 2005 to 2014 (APC = −2.35; 95% CI: −3.51–−1.18), a significant increase in 2014 to 2020 (APC = 4.97; 95% CI: 2.60–7.39), and no significant change in 2020 to 2023 (APC = −0.41; 95% CI: −4.95–4.36). See Table 1 and Figure 1.

### 3.3. Population differences

#### 3.3.1. Race and ethnicity

From 1999 to 2023, a total of 34,504 DU-related deaths were reported in the US, including 28,348 non-Hispanic White (82.1%), 2950 non-Hispanic Black (8.6%), 1791 Hispanic (5.2%), and 1315 individuals of other races (3.8%).

**Figure 2.**
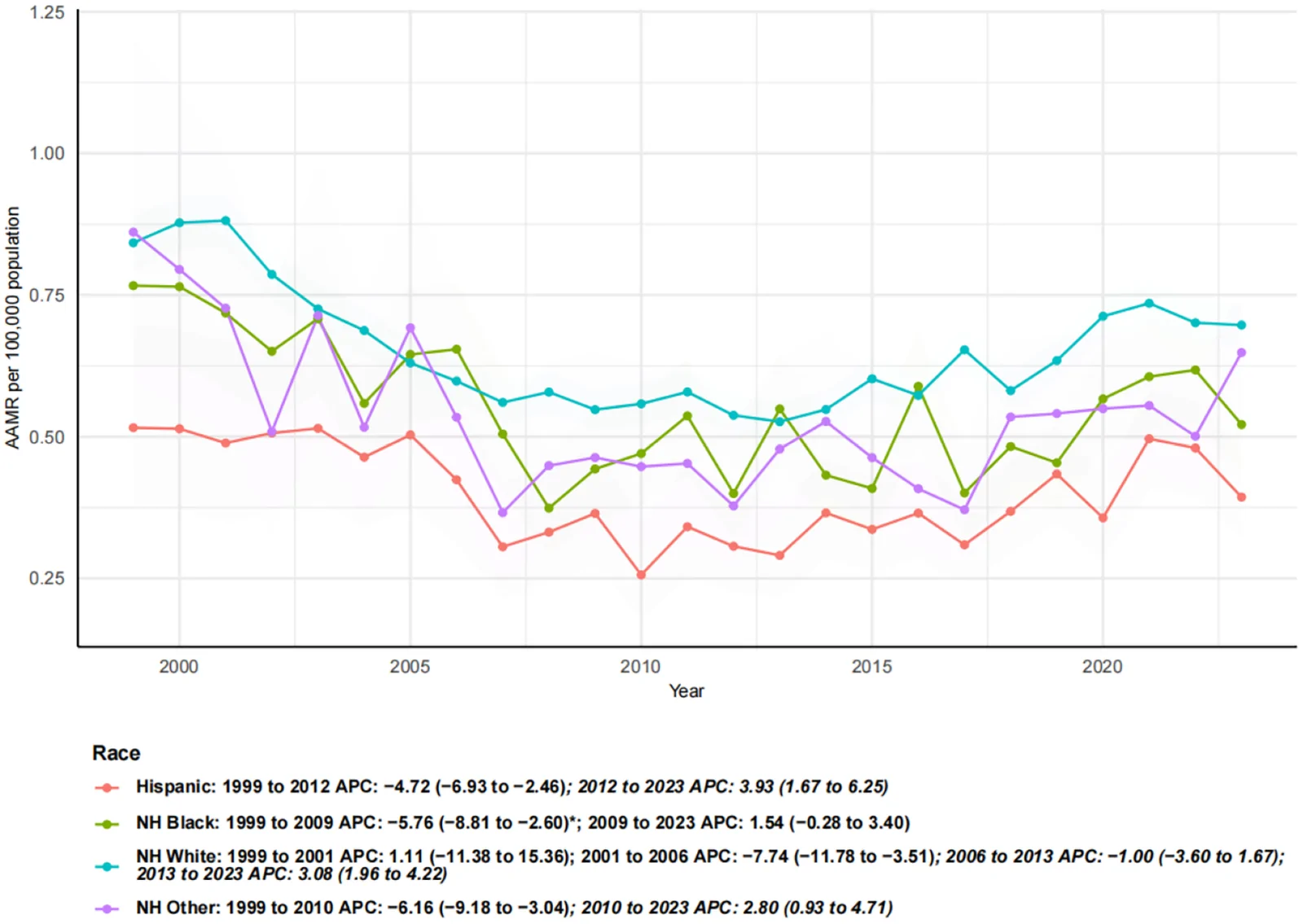
Trends in DU AAMRs stratified by race in the US, 1999–2023. AAMR = age-adjusted mortality rate, APC = annual perventage change, DU = duodenal ulcer, US = United States.

Hispanic: the number of deaths increased from 47 in 1999 to 124 in 2023, an increase of 163.83%. The AAMR decreased from 0.52 (95% CI: 0.37–0.70) in 1999 to 0.39 (95% CI: 0.32–0.47) in 2023. The AAPC was −0.85 (95% CI: −2.35–0.68), indicating no significant overall change in mortality. There was a significant decline in 1999 to 2012 (APC = −4.72; 95% CI: −6.93–−2.46), followed by a significant increase in 2012 to 2023 (APC = 3.93; 95% CI: 1.67–6.25). See Table 2 and Figure 2 for details.

**Table 2:** Trends in DU AAMRs stratified by race in the US, 1999–2023.

Race	Deaths_1999	Deaths_2023	Percent. change	AAMR_1999	AAMR_2023	AAPC (95% CI)
Hispanic	47	124	163.83	0.52 (0.37–0.70)	0.39 (0.32–0.47)	−0.85 (−2.35–0.68)
NH Black	121	155	28.10	0.77 (0.63–0.91)	0.52 (0.44–0.61)	−1.56 (−3.15–0.05)
NH White	1254	1386	10.53	0.84 (0.80–0.89)	0.70 (0.66–0.74)	−0.61 (−2.14–0.94)
NH Other	39	126	223.08	0.86 (0.60–1.20)	0.65 (0.53–0.76)	−1.41 (−3.06–0.27)

AAMR = age-adjusted mortality rate, AAPC = average annual perventage change, CI = confidence interval, DU = duodenal ulcer, US = United States.

Non-Hispanic White: the number of deaths increased from 1254 in 1999 to 1386 in 2023, an increase of 10.53%. The AAMR decreased from 0.84 (95% CI: 0.80–0.89) in 1999 to 0.70 (95% CI: 0.66–0.74) in 2023. The AAPC was −0.61 (95% CI: −2.14–0.94), indicating no significant overall change. By segments: 1999 to 2001 showed no significant change (APC = 1.11; 95% CI: −11.38–15.36), 2001 to 2006 showed a significant decline (APC = −7.74; 95% CI: −11.78–−3.51), 2006 to 2013 showed no significant change (APC = −1.00; 95% CI: −3.60–1.67), and 2013 to 2023 showed a significant increase (APC = 3.08; 95% CI: 1.96–4.22).

Non-Hispanic Black: the number of deaths increased from 121 in 1999 to 155 in 2023, an increase of 28.10%. The AAMR decreased from 0.77 (95% CI: 0.63–0.91) in 1999 to 0.52 (95% CI: 0.44–0.61) in 2023. The AAPC was −1.56 (95% CI: −3.15–0.05), indicating no significant overall change. By segments: 1999 to 2009 showed a significant decline (APC = −5.76; 95% CI: −8.81–−2.60), and 2009 to 2023 showed no significant change (APC = 1.54; 95% CI: −0.28–3.40).

Non-Hispanic Other: the number of deaths increased from 39 in 1999 to 126 in 2023, an increase of 223.08%. The AAMR decreased from 0.86 (95% CI: 0.60–1.20) in 1999 to 0.65 per 100,000 (95% CI: 0.53–0.76) in 2023. The AAPC was −1.41 (95% CI: −3.06 to 0.27), indicating no significant overall change. By segments: 1999 to 2010 showed a significant decline (APC = −6.16; 95% CI: −9.18–−3.04), and 2010 to 2023 showed a significant increase (APC = 2.80; 95% CI: 0.93–4.71).

### 3.4. Age

From 1999 to 2023, DU-related mortality varied markedly by age group. Overall, younger groups consistently had lower mortality, whereas older groups had substantially higher mortality with a notable rebound in the later study period.

**Figure 3.**
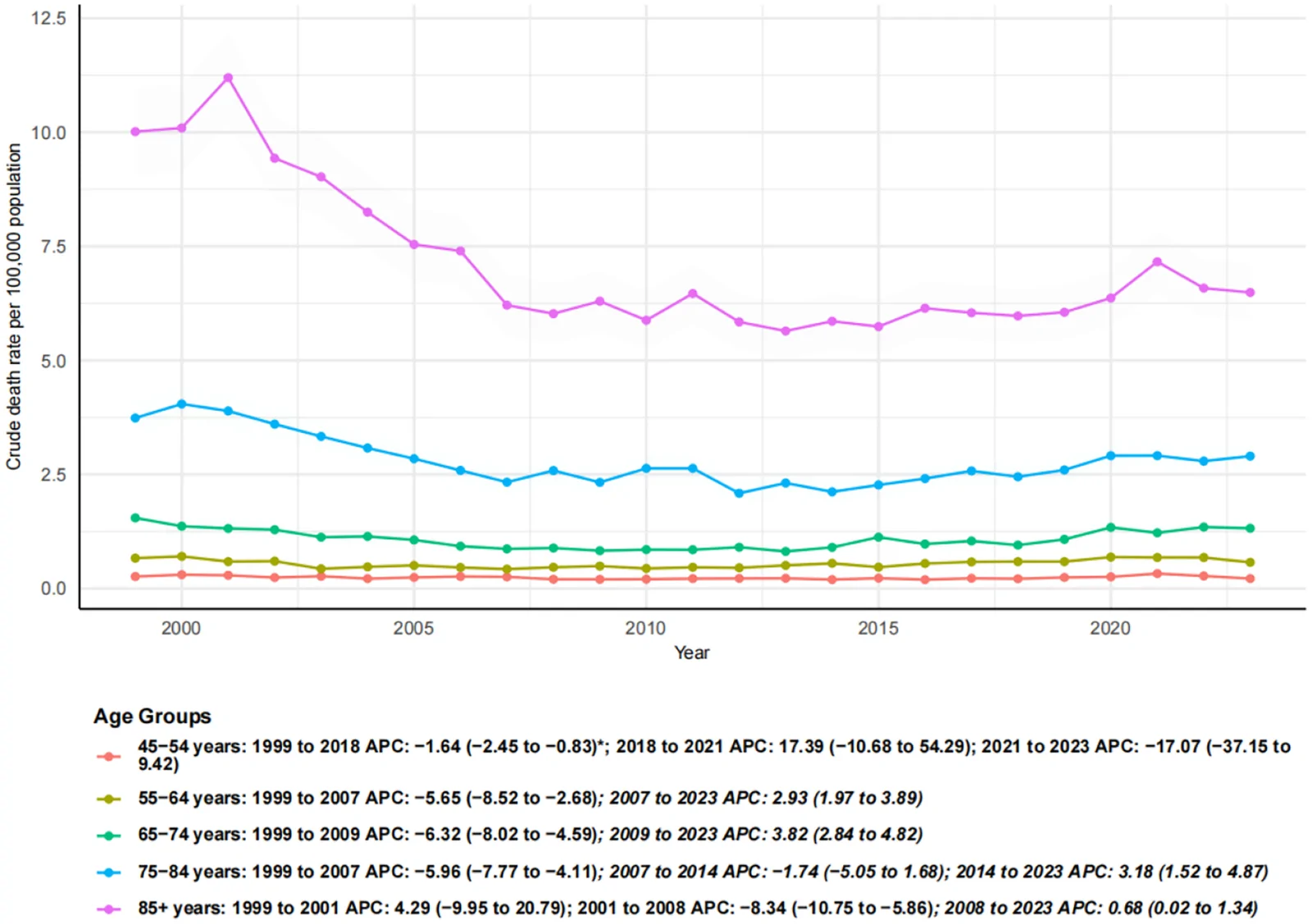
Trends in DU AAMRs stratified by age in the US, 19992023. AAMR = age-adjusted mortality rate, APC = annual perventage change, DU = duodenal ulcer, US = United States.

Ages 45 to 54: AAMR decreased from 0.26 (95% CI: 0.21–0.32) to 0.22 (95% CI: 0.17–0.27). AAPC, −0.86 (95% CI: −4.63–3.06); overall, not significant. Segments: 1999 to 2018 significant decline (APC = −1.64; 95% CI: −2.45–−0.83), 2018 to 2021 not significant (APC = 17.39; 95% CI: −10.68–54.29), 2021 to 2023 not significant (APC = −17.07; 95% CI: −37.15–9.42). See Table 3 and Figure 3.

**Table 3:** Trends in DU AAMRs stratified by age in the US, 1999–2023.

Age	Deaths_1999	Deaths_2023	Percent. change	AAMR_1999	AAMR_2023	AAPC (95% CI)
Ages 45–54	96	88	−8.33	0.26 (0.21–0.32)	0.22 (0.17–0.27)	−0.86 (−4.63–3.06)
55–64	158	240	51.90	0.66 (0.56–0.77)	0.57 (0.50–0.65)	−0.01 (−1.14–1.12)
65–74	285	458	60.70	1.55 (1.37–1.73)	1.32 (1.20–1.44)	−0.53 (−1.41–0.36)
75–84	457	533	16.63	3.74 (3.40–4.08)	2.90 (2.66–3.15)	−1.38 (−2.59–−0.14)*
85+	416	402	−3.37	10.01 (9.05–10.98)	6.49 (5.85–7.12)	−1.75 (−3.12–−0.37)*

AAMR = age-adjusted mortality rate, AAPC = average annual perventage change, CI = confidence interval, DU = duodenal ulcer, US = United States.

* indicates that the *P* value is statistically significant.

Ages 55 to 64: AAMR decreased from 0.66 (95% CI: 0.56–0.77) to 0.57 (95% CI: 0.50–0.65). AAPC, −0.01 (95% CI: −1.14–1.12); overall, not significant. Segments: 1999 to 2007 significant decline (APC = −5.65; 95% CI: −8.52–−2.68), 2007 to 2023 significant increase (APC = 2.93; 95% CI: 1.97–3.89).

Ages 65 to 74: AAMR decreased from 1.55 (95% CI: 1.37–1.73) to 1.32 (95% CI: 1.20–1.44). AAPC, −0.53 (95% CI: −1.41–0.36); overall, not significant. Segments: 1999 to 2009 significant decline (APC = −6.32; 95% CI: −8.02–−4.59), 2009 to 2023 significant increase (APC = 3.82; 95% CI: 2.84–4.82).

Ages 75 to 84: AAMR decreased from 3.74 (95% CI: 3.40–4.08) to 2.90 (95% CI: 2.66–3.15). AAPC, −1.38 (95% CI: −2.59–−0.14); significant decline overall. Segments: 1999 to 2007 significant decline (APC = −5.96; 95% CI: −7.77–−4.11), 2007 to 2014 not significant (APC = −1.74; 95% CI: −5.05–1.68), 2014 to 2023 significant increase (APC = 3.18; 95% CI: 1.52–4.87).

Ages ≥ 85: AAMR decreased from 10.01 (95% CI: 9.05–10.98) to 6.49 (95% CI: 5.85–7.12). AAPC, −1.75 (95% CI: −3.12–−0.37); significant decline overall. Segments: 2001 to 2008 sharp decline (APC = −8.34; 95% CI: −10.75–−5.86), 2008 to 2023 modest increase (APC = 0.68; 95% CI: 0.02–1.34).

### 3.5. Regional differences

#### 3.5.1. Census region–based differences

From 1999 to 2023, there were 11,242 deaths in the South, 9296 in the West, 7814 in the Midwest, and 6168 in the Northeast. All regions showed declines early in the study period, followed by subsequent increases.

**Figure 4.**
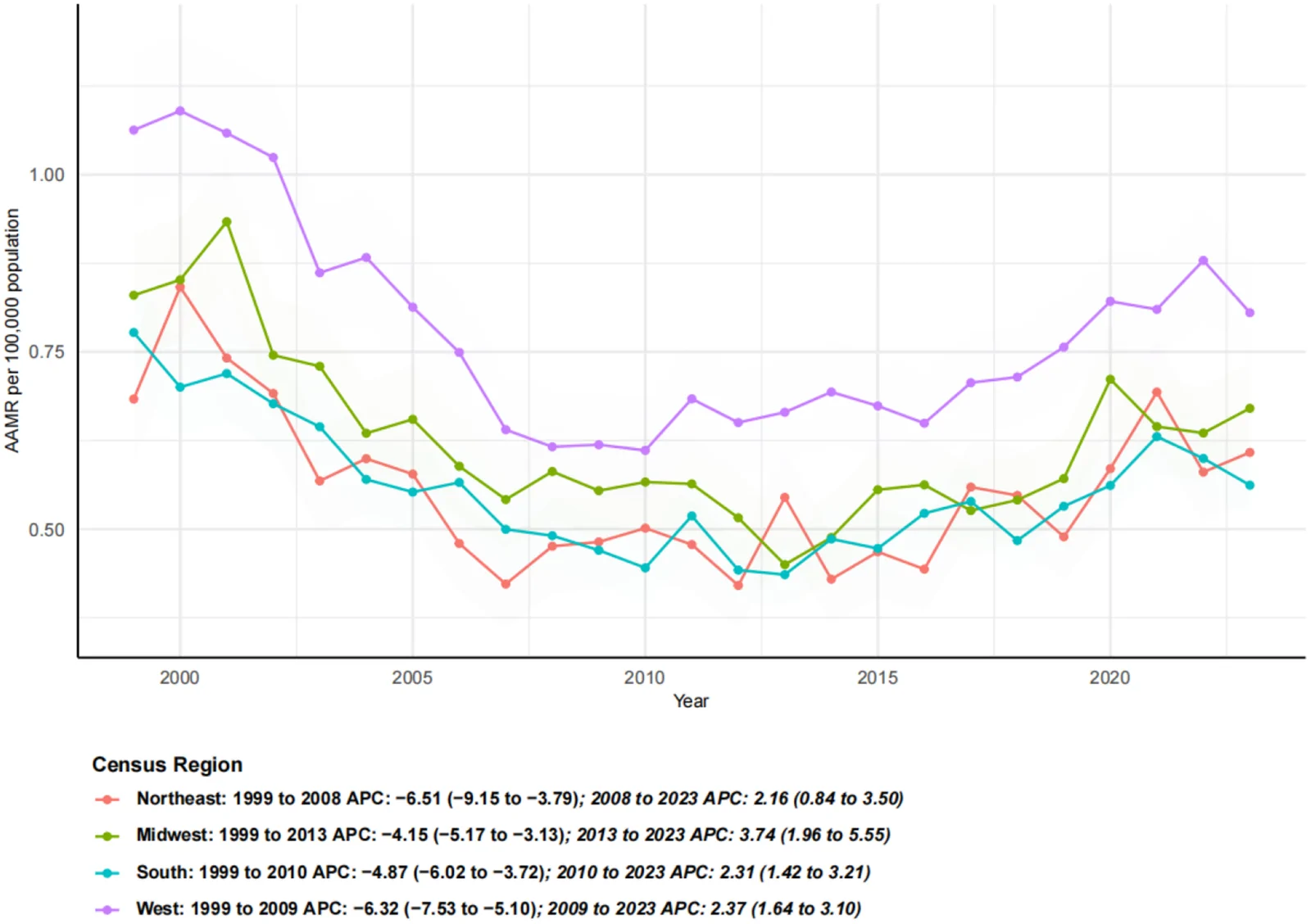
Trends in DU AAMRs stratified by census region in the US, 1999–2023. AAMR = age-adjusted mortality rate, APC = annual perventage change, DU = duodenal ulcer, US = United States.

Midwest: AAMR decreased from 0.83 (95% CI: 0.74–0.92) to 0.67 (95% CI: 0.60–0.74). AAPC = −0.94 (95% CI: −1.82–−0.05); overall declining trend. Segments: 1999 to 2013 significant decline (APC = −4.15; 95% CI: −5.17–−3.13), 2013 to 2023 significant increase (APC = 3.74; 95% CI: 1.96–5.55). See Table 4 and Figure 4.

**Table 4:** Trends in DU AAMRs stratified by census region in the US, 1999–2023.

Measure	Deaths_1999	Deaths_2023	Percent. change	AAMR_1999	AAMR_2023	AAPC (95% CI)
Northeast	264	307	16.29	0.68 (0.60–0.77)	0.61 (0.54–0.68)	−1.18 (−2.43–0.08)
Midwest	363	398	9.64	0.83 (0.74–0.92)	0.67 (0.60–0.74)	−0.94 (−1.82–−0.05)*
South	474	596	25.74	0.78 (0.71–0.85)	0.56 (0.52–0.61)	−1.05 (−1.72–−0.37)*
West	369	493	33.60	1.06 (0.95–1.17)	0.81 (0.73–0.88)	−1.35 (−1.98–−0.71)*

AAMR = age-adjusted mortality rate, AAPC = average annual perventage change, CI = confidence interval, DU = duodenal ulcer, US = United States.

* indicates that the *P* value is statistically significant.

West: AAMR decreased from 1.06 (95% CI: 0.95–1.17) to 0.81 (95% CI: 0.73–0.88). AAPC = −1.35 (95% CI: −1.98–−0.71); overall declining trend. Segments: 1999 to 2009 significant decline (APC = −6.32; 95% CI: −7.53–−5.10), 2009 to 2023 significant increase (APC = 2.37; 95% CI: 1.64–3.10).

South: AAMR decreased from 0.78 (95% CI: 0.71–0.85) to 0.56 per 100 000 (95% CI: 0.52–0.61). AAPC = −1.05 (95% CI: −1.72–−0.37); overall declining trend. Segments: 1999 to 2010 significant decline (APC = −4.87; 95% CI: −6.02–−3.72), 2010 to 2023 significant increase (APC = 2.31; 95% CI: 1.42–3.21).

Northeast: AAMR decreased from 0.68 (95% CI: 0.60–0.77) to 0.61 (95% CI: 0.54–0.68). AAPC = −1.18% (95% CI: −2.43–0.08); overall, not significant. Segments: 1999 to 2008 significant decline (APC = −6.51; 95% CI: −9.15–−3.79), 2008 to 2023 significant increase (APC = 2.16; 95% CI: 0.84–3.50).

#### 3.5.2. States

Between 1999 and 2023, trends in the AAMR of DUs varied significantly across US states. Most states exhibited a declining trend, with California showing a particularly pronounced decrease (AAPC = −1.83; 95% CI: −3.63–0.00), where the AAMR decreased from 1.23 (95% CI: 1.07–1.39) in 1999 to 0.74 (95% CI: 0.64–0.84) in 2023. Georgia (AAPC = −2.27; 95% CI: 4.22–0.27), Illinois (AAPC = −2.22; 95% CI: −4.30–−0.11), and Michigan (AAPC = −1.75; 95% CI: −3.46–−0.01) also exhibited significant downward trends. In contrast, Washington (AAPC = 0.21; 95% CI: −1.83–2.29) and Wisconsin (AAPC = 0.03; 95% CI: −0.87–0.93) showed no significant decline in mortality rates, with AAMR remaining largely stable throughout the observation period. Pennsylvania showed no significant change in mortality (AAPC = −0.02; 95% CI: −2.00–2.00), with a 2023 AAMR of 0.70 (95% CI: 0.55–0.88), similar to its 1999 level. See Table 5.

**Table 5:** The trend of AAMR rate of DU in each state of the US from 1999 to 2023 (by state).

State	Deaths_ALL	AAMR_1999	AAMR_2023	AAPC (95% CI)
California	4743	1.23 (1.07–1.39)	0.74 (0.64–0.84)	−1.83 (−3.63–0.00)*
Florida	2176	0.75 (0.61–0.92)	0.56 (0.46–0.67)	−1.18 (−2.50–0.16)
Georgia	764	0.86 (0.60–1.19)	0.46 (0.32–0.64)	−2.27 (−4.22–−0.27)*
Illinois	1257	0.94 (0.74–1.19)	0.59 (0.46–0.76)	−2.22 (−4.30–−0.11)*
Michigan	1072	0.73 (0.53–0.98)	0.60 (0.45–0.79)	−1.75 (−3.46–−0.01)*
New York	2365	0.79 (0.63–0.94)	0.63 (0.51–0.75)	−1.22 (−2.49–0.07)
North Carolina	985	0.79 (0.56–1.08)	0.73 (0.56–0.94)	−0.77 (−2.06–0.53)
Ohio	1490	0.94 (0.73–1.19)	0.71 (0.55–0.90)	−0.55 (−2.26–1.19)
Pennsylvania	1437	0.63 (0.48–0.82)	0.70 (0.55–0.88)	−0.02 (−2.00–2.00)
Texas	2034	0.79 (0.63–0.99)	0.56 (0.45–0.67)	−0.43 (−2.02–1.19)
Washington	1043	0.88 (0.60–1.25)	1.13 (0.87–1.44)	0.21 (−1.83–2.29)
Wisconsin	767	0.83 (0.56–1.18)	0.78 (0.56–1.07)	0.03 (−0.87–0.93)

AAMR = age-adjusted mortality rate, AAPC = average annual perventage change, CI = confidence interval, DU = duodenal ulcer, US = United States.

* indicates that the *P* value is statistically significant.

#### 3.5.3. Metropolitan vs nonmetropolitan

**Figure 5.**
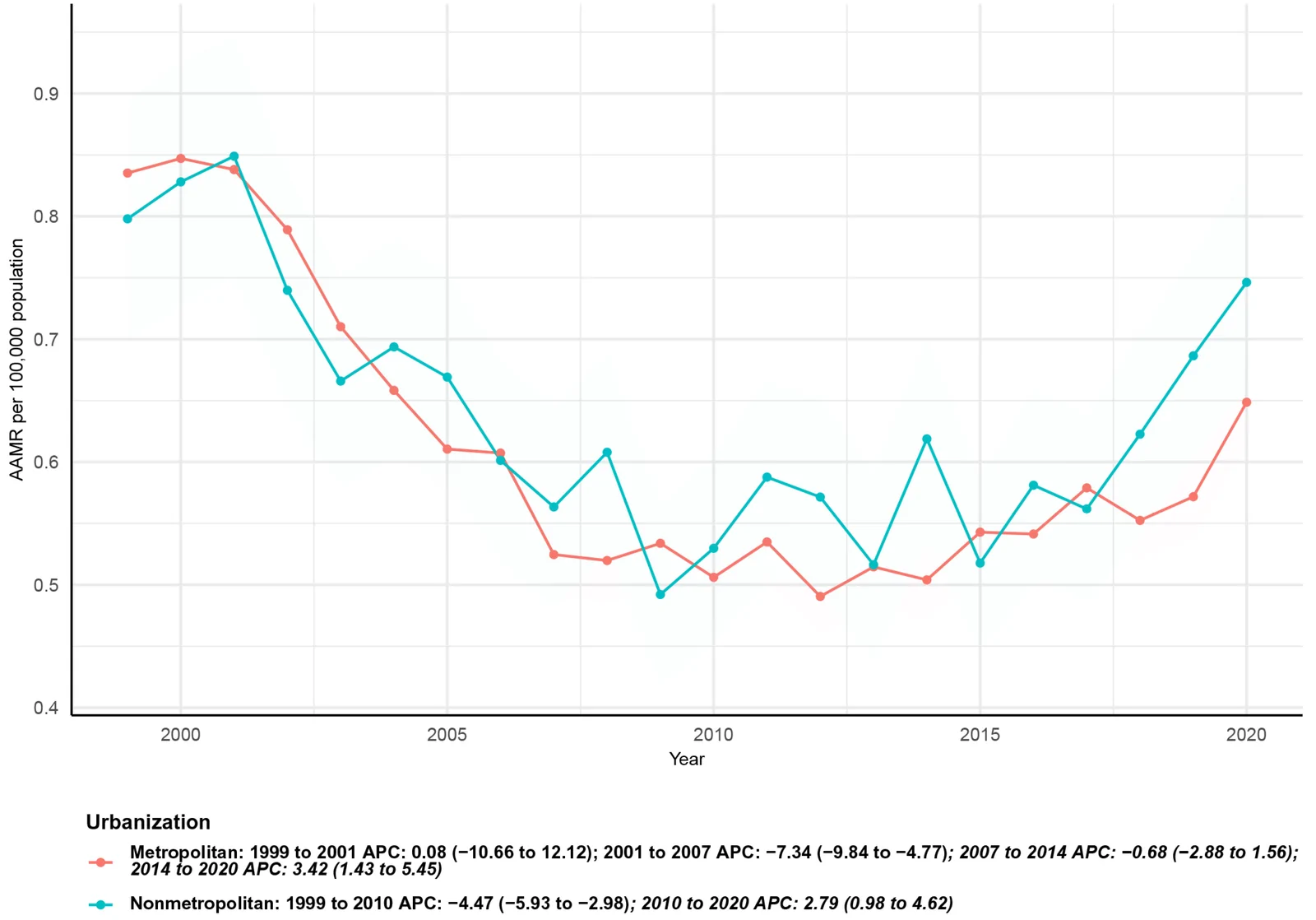
Trends in DU age AAMRs stratified by urban vs rural in the US, 1999–2023. AAMR = age-adjusted mortality rate, APC = annual perventage change, DU = duodenal ulcer, US = United States.

From 1999 to 2023, there were 23,827 deaths in metropolitan areas and 5263 deaths in nonmetropolitan areas. Throughout the study period, AAMRs were consistently higher in nonmetropolitan than in metropolitan areas: in nonmetropolitan areas, AAMR decreased from 0.80 (95% CI: 0.70–0.89) to 0.75 (95% CI: 0.66–0.83); in metropolitan areas, from 0.84 (95% CI: 0.79–0.88) to 0.65 (95% CI: 0.61–0.68). For overall trends, the metropolitan AAPC was −1.43 (95% CI: −2.84–0.01), indicating no significant overall change; the nonmetropolitan AAPC was −1.08 (95% CI: −2.15–0.00), indicating an overall declining trend.By segments, metropolitan areas showed a significant decline in 2001 to 2007 (APC = −7.34; 95% CI: −9.84–−4.77) and a significant increase in 2014 to 2020 (APC = 3.42; 95% CI: 1.43–5.45). Nonmetropolitan areas showed a significant decline in 1999 to 2010 (APC = −4.47; 95% CI: −5.93–−2.98) and a significant increase in 2010 to 2020 (APC = 2.79; 95% CI: 0.98–4.62).See Table 6 and Figure 5 for more details.

**Table 6:** Trends in DU AAMRs stratified by urban vs rural in the US, 1999–2023.

Measure	Deaths_1999	Deaths_2023	Percent.change	AAMR_1999	AAMR_2023	AAPC (95% CI)
Metropolitan	1206	1484	18.73	0.84 (0.79–0.88)	0.65 (0.61–0.68)	−1.43 (−2.84–0.01)
Nonmetropolitan	264	310	17.42	0.80 (0.70–0.89)	0.75 (0.66–0.83)	−1.08 (−2.15–0.00)*

The classification of urbanization levels in 2023 by AAMR uses data from 2020, and the the calculation time range for AAPC is from 1999 to 2023.

AAMR = age-adjusted mortality rate, AAPC = average annual perventage change, CI = confidence interval, DU = duodenal ulcer, US = United States.

* indicates that the *P* value is statistically significant.

## 4. Discussion

Using US CDC WONDER data from 1999 to 2023, we characterized long-term patterns in DU-related mortality. We identified a notable public health paradox: although the overall AAMR declined, multiple groups showed a rebound beginning in the 2010s. This “decline-then-rise” pattern appeared across sexes, racial and ethnic groups, and age strata, challenging the assumption that mortality would continue to fall with wider access to effective therapies. These results suggest that DU mortality reflects the joint influence of medical advances, demographic shifts, social determinants, and emerging risks (e.g., medication exposures and antimicrobial resistance).

From 1999 to 2023, the AAMR decreased from 0.83 to 0.64 per 100 000, and the AAPC was −1.32% (not statistically significant). The early decline (1999–2009) likely reflects population-level benefits following the recognition of *H pylori* as the primary cause of peptic ulcer disease, the shift from acid suppression alone to eradication-focused management, the widespread use of PPIs, and advances in endoscopic hemostasis. Joinpoint analyses, however, indicate an inflection around 2009 to 2013, with significant increases in several subgroups through 2013 to 2023, implying diminishing returns from earlier interventions and the emergence of new pressures. Plausible contributors include: rising *H pylori* resistance: increasing clarithromycin resistance (with rates exceeding 20–30% in some settings) may undermine first-line triple therapy and elevate risks of recurrence and complications^[12]^; NSAID/aspirin exposure: independent of *H pylori*, long-term use of NSAIDs and aspirin has expanded with population aging and management of chronic conditions^[13,14]^; behavioral and social changes: smoking, heavy alcohol use, psychosocial stress, and potential effects of the opioid crisis on gastrointestinal function may contribute^[15]^; cohort effects and risk concentration: successful *H pylori* control among younger adults may have shifted the remaining burden toward older patients with multimorbidity and lower responsiveness to treatment, consistent with our age-stratified findings; system shocks: health system strain during public health emergencies (e.g., deferred procedures, delayed emergency care) has been associated with excess mortality in other diseases and may similarly affect DU outcomes.^[16]^

Men consistently had higher DU mortality than women, aligning with prior global literature and potentially reflecting biological (e.g., hormonal influences on acid secretion and mucosal protection) and behavioral factors (higher historical rates of smoking and alcohol use).^[17]^ Importantly, trends diverged over time: after a rapid decline in 1999 to 2007, male mortality rose significantly in 2013 to 2023. Female mortality, although not significantly declining overall, also increased during 2014 to 2020. These parallel upturns suggest shared drivers across sexes; possibilities include increased NSAID use among women (e.g., for migraine and arthritis) or shifts in care-seeking and adherence patterns. Further work is needed to clarify mechanisms.

While non-Hispanic Black individuals showed the largest absolute decline in AAMR from 1999 to 2023 (0.77–0.52), the decrease concentrated in 1999 to 2009 and plateaued thereafter. Hispanic and “other” groups experienced significant increases after early declines in the 2010s. These patterns highlight persistent health inequities. Likely contributors include disparities in access to primary and specialty care, timely endoscopy, and high-quality management^[18]^; socioeconomic disadvantage that correlates with higher *H pylori* prevalence, delayed diagnosis, and lower adherence^[19]^; and differences in culture, language, and health literacy that influence recognition and care. Although non-Hispanic White individuals account for most deaths (82.1%), their mortality also rose after 2013, indicating that the rebound is widespread and not confined to minority groups, while still necessitating targeted, culturally responsive interventions.

Age-stratified results indicate an increasing concentration of DU mortality among older adults and the most pronounced rebound in these groups. Mortality remained low and relatively stable for ages 45 to 54 and 55 to 64, consistent with the durable benefits of *H pylori* eradication in younger and midlife cohorts. In contrast, ages 55 to 64 and 65 to 74 exhibited a decline followed by sustained increases, with ages 65 to 74 showing the steepest post-2009 growth. This cohort overlaps with the “baby boomer” generation, who may have had higher historic *H pylori* exposure^[20]^ and currently more chronic NSAID/aspirin use. Mortality levels were highest in ages 75 to 84 and ≥ 85. Despite overall declines by AAPC, joinpoint segments showed renewed increases (75–84 after 2014; ≥ 85 after 2008), potentially reflecting multimorbidity, altered drug metabolism, atypical presentations that delay diagnosis, lower tolerance to bleeding, and more conservative clinical decision-making in advanced age.^[21–24]^

All 4 census regions (Midwest, West, South, Northeast) exhibited the “decline-then-rise” pattern, implying national drivers. Baseline levels and trajectories varied (e.g., highest AAMR in the West in 1999 vs lowest in the South in 2023), potentially due to regional differences in demographics, socioeconomic context, health system capacity, and primary care quality. State-level results further refine geographic targets (e.g., trend reversals in California, Texas, and Florida; more recent acceleration in Pennsylvania and Ohio; persistent increases in Washington), suggesting state-specific policies or coverage differences may play a role. Urban–rural analyses showed persistently higher mortality in nonmetropolitan areas, consistent with a broader “rural health disadvantage.” The gap persisted after age standardization, suggesting contributions from specialist shortages, longer travel times for emergency and endoscopic care, lower insurance coverage, higher smoking prevalence, and limited health information resources.^[25]^ The significant rise in nonmetropolitan mortality during 2010 to 2020 underscores the vulnerability of rural systems to national shocks.

Clinicians should recognize that DU mortality risk is increasingly concentrated among older adults with multimorbidity and ulcerogenic medications and consider proactive gastroprotection (e.g., PPI co-therapy) in high-risk patients. Given rising resistance, susceptibility-guided or locally informed *H pylori* eradication strategies should be prioritized over empiric regimens with declining efficacy. For rural and low-income populations, risk counseling and safer NSAID/aspirin prescribing are essential, alongside public health efforts to improve access to diagnostic endoscopy and urgent care. Targeted screening and eradication programs in high-risk communities, coupled with stronger primary care and referral pathways, may reduce mortality. Public education on over-the-counter NSAIDs and their risks is also warranted.

Strengths include the use of a nationwide mortality registry with large population coverage and long time span, enabling detection of long-term trends and joinpoints. Limitations include potential misclassification inherent in death certificates; a lack of distinction between DU and gastric ulcer, rendering results applicable to peptic ulcer disease overall rather than DU alone; and the absence of individual-level clinical data (e.g., *H pylori* status, medication use, endoscopy, comorbidities), precluding direct testing of hypothesized mechanisms (e.g., resistance, polypharmacy). Reporting biases may exist, and cross-stratified analyses (e.g., age-by-race) were not performed. Socioeconomic variables (income, education, insurance) are unavailable in CDC WONDER, limiting the assessment of confounding. Findings from the US may not generalize to other settings. As an ecological study, causal inference at the individual level is not possible.

## 5. Conclusion

US DU mortality declined to a nadir in the early 21st century but rebounded broadly after approximately 2010, most prominently among older adults, selected minority groups, and rural residents. The burden appears to be shifting toward high-risk populations, marking a new stage for prevention and control. Addressing persistent inequities will require moving beyond one-size-fits-all approaches toward risk-stratified, targeted strategies and deeper investigation of drivers such as *H pylori* resistance, polypharmacy, and social determinants. Coordinated efforts across clinical practice, public health, and policy are needed to reverse adverse trends and ensure that the benefits of medical progress are equitably distributed.

## Author contributions

**Conceptualization:** Ming-Jie Jia.

**Data curation:** Ming-Jie Jia.

**Validation:** Bo-Ying Wu.

**Visualization:** Bo-Ying Wu.

**Writing – original draft:** Feng Jiang.

**Writing – review & editing:** Feng Jiang.

## Correction

The funding information has now been updated online from “This study was supported by the National Natural Science Foundation of China (Grant No. 82260901)” to This study was supported by the National Natural Science Foundation of China (Grant Nos. 82560921 and 82260901).

## References

[1] Samloff IM. Peptic ulcer: the many proteinases of aggression. Gastroenterology. 1989;96(2 Pt 2 Suppl):586–95. 10.1016/s0016-5085(89)80054-x 2642445

[2] Marshall BJ Warren JR. Unidentified curved bacilli in the stomach of patients with gastritis and peptic ulceration. Lancet. 1984;1:1311–5. 10.1016/s0140-6736(84)91816-6 6145023

[3] Lanas A Chan FKL. Peptic ulcer disease. Lancet. 2017;390:613–24. 10.1016/S0140-6736(16)32404-7 28242110

[4] Lu CL Chang SS Wang SS Chang FY Lee SD. Silent peptic ulcer disease: frequency, factors leading to "silence," and implications regarding the pathogenesis of visceral symptoms. Gastrointest Endosc. 2004;60:34–8. 10.1016/s0016-5107(04)01311-2 15229422

[5] Brown TJ Hooper L Elliott RA. A comparison of the cost-effectiveness of five strategies for the prevention of non-steroidal anti-inflammatory drug-induced gastrointestinal toxicity: a systematic review with economic modelling. Health Technol Assess. 2006;10:iii–v, xi. 10.3310/hta10380 17018227

[6] Nguyen TNM Sha S Chen LJ Holleczek B Brenner H Schöttker B. Strongly increased risk of gastric and duodenal ulcers among new users of low-dose aspirin: results from two large cohorts with new-user design. Aliment Pharmacol Ther. 2022;56:251–62. 10.1111/apt.17050 35621052

[7] Kurata JH Elashoff JD Haile BM Honda GD. A reappraisal of time trends in ulcer disease: factors related to changes in ulcer hospitalization and mortality rates. Am J Public Health. 1983;73:1066–72. 10.2105/ajph.73.9.1066 PMC1651068 6881404

[8] Achara KE Iyayi IR Erinne OC. Trends and Patterns in Obesity-Related Deaths in the US (2010–2020): a comprehensive analysis using centers for disease control and prevention wide-ranging online data for epidemiologic research (CDC WONDER) Data. Cureus. 2024;1:10. 10.7759/cureus.68376 PMC11443986 39355487

[9] Rethy L Shah NS Paparello JJ Lloyd-Jones DM Khan SS. Trends in hypertension-related cardiovascular mortality in the United States, 2000 to 2018. Hypertension. 2020;76:e23–5. 10.1161/HYPERTENSIONAHA.120.15153 PMC9390965 32654559

[10] CDC. National Center for Health Statistics. 2024. https://www.cdc.gov/nchs/data-analysis-tools/urban-rural.html. Accessed December 9, 2024

[11] Ingram DD Franco SJ. NCHS Urban-Rural classification scheme for counties. Vital Health Stat 2. 2013;2014:1–73. Return to ref 12 in article. 24776070

[12] Malfertheiner P Megraud F Rokkas T; European Helicobacter and Microbiota Study group. Management of <i>Helicobacter pylori</i> infection: the Maastricht VI/Florence consensus report. Gut. 2022;71:1724–62. 10.1136/gutjnl-2022-327745 35944925

[13] GBD 2015 Disease and Injury Incidence and Prevalence Collaborators. Global, regional, and national incidence, prevalence, and years lived with disability for 310 diseases and injuries, 1990-2015: a systematic analysis for the Global Burden of Disease Study 2015. Lancet. 2016;388:1545–602. 10.1016/S0140-6736(16)31678-6 PMC5055577 27733282

[14] Blot WJ McLaughlin JK. Over the counter non-steroidal anti-inflammatory drugs and risk of gastrointestinal bleeding. J Epidemiol Biostat. 2000;5:137–42. 10890286

[15] Brock C Olesen SS Olesen AE Frøkjaer JB Andresen T Drewes AM. Opioid-induced bowel dysfunction: pathophysiology and management. Drugs. 2012;72:1847–65. 10.2165/11634970-000000000-00000 22950533

[16] COVIDSurg Collaborative. Elective surgery cancellations due to the COVID-19 pandemic: global predictive modelling to inform surgical recovery plans. Br J Surg. 2020;107:1440–9. 10.1002/bjs.11746 PMC7272903 32395848

[17] Ford AC Marwaha A Sood R Moayyedi P. Global prevalence of, and risk factors for, uninvestigated dyspepsia: a meta-analysis. Gut. 2015;64:1049–57. 10.1136/gutjnl-2014-307843 25147201

[18] Ajayi Sotubo O. A perspective on health inequalities in BAME communities and how to improve access to primary care. Future Healthc J. 2021;8:36–9. 10.7861/fhj.2020-0217 PMC8004339 33791458

[19] Malcolm CA MacKay WG Shepherd A Weaver LT. Helicobacter pylori in children is strongly associated with poverty. Scott Med J. 2004;49:136–8. 10.1177/003693300404900406 15648706

[20] Grad YH Lipsitch M Aiello AE. Secular trends in Helicobacter pylori seroprevalence in adults in the United States: evidence for sustained race/ethnic disparities. Am J Epidemiol. 2012;175:54–9. 10.1093/aje/kwr288 PMC3244610 22085628

[21] Blatchford O Murray WR Blatchford M. A risk score to predict need for treatment for upper-gastrointestinal haemorrhage. Lancet. 2000;356:1318–21. 10.1016/S0140-6736(00)02816-6 11073021

[22] Kemppainen H Räihä I Sourander L. Clinical presentation of peptic ulcer in the elderly. Gerontology. 1997;43:283–8. 10.1159/000213864 9309418

[23] Baty V. Helicobacter pylori infection management in elderly patients with oral anticoagulation. Am J Med. 2018;131:e221. 10.1016/j.amjmed.2017.04.023 29673497

[24] Fransvea P Costa G Lepre L; ERASO (Elderly Risk Assessment and Surgical Outcome) Collaborative Study Group. Laparoscopic repair of perforated peptic ulcer in the elderly: an interim analysis of the FRAILESEL Italian multicenter prospective cohort study. Surg Laparosc Endosc Percutan Tech. 2020;31:2–7. 10.1097/SLE.0000000000000826 32675754

[25] Carmichael H Samuels JM Velopulos CG Jones EL. Geographic distribution of colonoscopy providers in the United States: an analysis of medicare claims data. Surg Endosc. 2022;36:7673–8. 10.1007/s00464-022-09083-3 35729404

